# Calculation of Splicing Potential from the Alternative Splicing Mutation Database

**DOI:** 10.1186/1756-0500-1-4

**Published:** 2008-02-26

**Authors:** Jason M Bechtel, Preeti Rajesh, Irina Ilikchyan, Ying Deng, Pankaj K Mishra, Qi Wang, Xiaochun Wu, Kirill A Afonin, William E Grose, Ye Wang, Sadik Khuder, Alexei Fedorov

**Affiliations:** 1 Program in Bioinformatics and Proteomics/Genomics, University of Toledo Health Science Campus, Toledo, Ohio 43614, USA; 2 Dept. of Medical Microbiology and Immunology, University of Toledo Health Science Campus, Toledo, Ohio 43614, USA; 3 Dept. of Basic Pharmaceutical Sciences, South Carolina College of Pharmacy, University of South Carolina, Columbia, South Carolina 29208, USA; 4 Department of Biological Sciences, Bowling Green State University, Bowling Green, Ohio 43403, USA; 5 College of Engineering, University of Toledo, Toledo, Ohio 43606, USA; 6 Dept. of Medicine, University of Toledo Health Science Campus, Toledo, Ohio 43614, USA

## Abstract

**Background:**

The Alternative Splicing Mutation Database (ASMD) presents a collection of all known mutations inside human exons which affect splicing enhancers and silencers and cause changes in the alternative splicing pattern of the corresponding genes.

**Findings:**

An algorithm was developed to derive a Splicing Potential (SP) table from the ASMD information. This table characterizes the influence of each oligonucleotide on the splicing effectiveness of the exon containing it. If the SP value for an oligonucleotide is positive, it promotes exon retention, while negative SP values mean the sequence favors exon skipping. The merit of the SP approach is the ability to separate splicing signals from a wide range of sequence motifs enriched in exonic sequences that are attributed to protein-coding properties and/or translation efficiency. Due to its direct derivation from observed splice site selection, SP has an advantage over other computational approaches for predicting alternative splicing.

**Conclusion:**

We show that a vast majority of known exonic splicing enhancers have highly positive cumulative SP values, while known splicing silencers have core motifs with strongly negative cumulative SP values. Our approach allows for computation of the cumulative SP value of any sequence segment and, thus, gives researchers the ability to measure the possible contribution of any sequence to the pattern of splicing.

## Background

One of the key regulators of alternative splicing is a large variety of short sequence motifs inside exons known as exonic splicing enhancers (ESE) and exonic splicing silencers (ESS). These regulatory sequences have been characterized by several experimental techniques [1-5] and also by different computational approaches [6-14]. Despite this progress, one still can not predict predisposition to alternative splicing from genomic data. In this respect, a set of mutations known to be associated with alternative splicing effects (reviewed by [15,5]) is a valuable raw material for the investigation of the fine regulation of splicing. A novel database of these mutations, named the Alternative Splicing Mutation Database (ASMD), is described in the accompanying paper [16]. The ASMD represents a collection of human exon sequences with internal mutations that change the balance of alternatively spliced mRNA isoforms or cause the appearance of new mRNA isoforms. The ASMD includes only those mutations that change exonic enhancers and silencers and does not encompass those that change splice sites. Here we present a novel statistical approach for processing ASMD mutational datasets, converting them into a table of "Splicing Potential" (SP) values for every possible short oligonucleotide. If the SP value for an oligonucleotide is positive, it promotes exon retention, while negative SP values mean the sequence favors exon skipping. SP appears to be a valuable tool for evaluating the influence of a given sequence on splicing, for finding and testing putative ESE and ESS motifs, and for predicting the effect of a given mutation on splicing.

## Algorithm for calculation of Splicing Potential

Our SP algorithm processes all oligonucleotides that appear and disappear in the mutations described in the ASMD. Due to the limited size of the current ASMD dataset, we only calculate SP values for triplets. For example, the mutation (G -> T) in the 14^th ^exon of the gene *BRCA1 *(entry '10asmd') occurs in the exonic region *gctGagt *-> *gctTagt *(mutation site is in the middle and is shown in capital letters). This mutation generates three new triplets (*ctT*, *tTa*, and *Tag*) and, at the same time, eliminates three triplets from the wild-type sequence (*ctG*, *tGa*, and *Gag*). The splicing effect of this mutation is SE = -1, meaning that this mutation causes the 14^th ^exon to be skipped in all gene transcripts. Because the wild-type triplets *ctG, tGa*, and *Gag *strengthen splicing of the exon, the algorithm increases their potential values by the value SP_*i *_= log_10_(*w*), where *w *= 1+abs(SE) and index *i *is the case identifier. In this example, SE is equal to -1, and thus *w *= 2. In our algorithm, *w *is simply the weight factor that awards more impact to those mutations that cause more dramatic changes in splicing patterns. In addition, because of the mutant triplets (*ctT*, *tTa*, and *Tag*) weaken the splicing of the exon, the algorithm decreases their SP values by the same value of SP_*i *_= -log_10_(*w*). The final potential value for a particular triplet *xyz *is the sum of SP_*i*_(*xyz*) for all cases in the ASMD where this triplet appears/disappears due to mutations. Finally, to make the SP values independent of the ASMD sample size, we normalize them by the standard deviation of SP values (σ_SP_). Thus, final SP values are calculated by the formula:

(1) SP(*xyz*) = sum(SP_i_(*xyz*))/σ_SP_.

We compared SP(*xyz*) with the coding potential, CP(*xyz*), for the triplet *xyz*, which was calculated by one of the simplest forms using equation:

(2) CP(*xyz*) = log_10_(F_c_(*xyz*)/F_i_(*xyz*))/σ_CP_,

where F_c_(*xyz*) is the frequency of the triplet *xyz *inside coding exonic regions, and F_i_(*xyz*) is the frequency of *xyz *inside introns. Throughout this study we multiplied all SP and CP values by 0.243, the σ_CP _for the entire sample of non-redundant human genes. If CP(*xyz*) has a positive value, the *xyz *triplet is more abundant in exons versus introns. When CP(*xyz*) is negative, the opposite is true and *xyz *is more abundant in introns. There are several much more advanced formulas for computing CP, which take into account additional information such as reading frames, exon length, overall genome composition, etc. [17,18]. Usually these approaches use advanced statistics, such as Markov models. However, for proper and adequate comparison of our initial data of SP values versus CP values we deliberately used formula (2). Both formula (2) and (1) do not account for reading frames and other genomic peculiarities. Such restrictions are appropriate for the limited size of the current ASMD dataset.

The SP and CP values for all 64 triplets are shown in Table 1. The more positive the SP value of the triplet, the more frequently its appearance is associated with retention of the exon containing it. Conversely, the more negative the SP value, the more frequently its inclusion is associated with exon skipping. Table 1 reveals a considerable Pearson correlation (r = 0.59) between coding potential (CP) and splicing potential (SP) values of triplets. A majority of triplets with positive CP values (meaning that their frequency in exons is greater than introns) also have positive SP values, while triplets with negative CP values frequently have negative SP values. However, the SP and CP values of a given triplet can differ significantly (for instance, see triplets *ccg, ccc, ggg, taa, att*).

**Table 1:** List of 64 triplets and their CP and SP values calculated for human genes.

Triplet	CP	SP_n_	Triplet	CP	SP_n_	Triplet	CP	SP_n_	Triplet	CP	SP_n_
CGC	0.57	0.19	GCA	0.14	-0.03	GTC	0.05	0.01	CTT	-0.13	-0.11
CGG	0.56	0.30	GCT	0.13	0.06	GTG	0.03	-0.08	CTA	-0.13	-0.03
CCG	0.55	-0.02	CTG	0.13	0.03	AAC	0.03	0.06	TCT	-0.15	-0.03
GCG	0.55	0.51	CCA	0.12	-0.03	GAT	0.02	0.25	TTG	-0.17	-0.19
CGA	0.51	0.45	GAG	0.12	0.15	CTC	0.02	0.14	TGT	-0.17	-0.40
ACG	0.43	0.18	TGC	0.12	-0.06	ATC	0.02	0.03	AAA	-0.18	-0.05
TCG	0.40	0.56	GAA	0.11	0.18	ATG	0.01	-0.13	GTT	-0.21	-0.23
CGT	0.30	0.28	TGG	0.10	0.17	ACA	0.00	-0.13	AAT	-0.25	0.08
GCC	0.24	0.02	AAG	0.10	0.26	TGA	0.00	-0.09	GTA	-0.26	-0.21
GAC	0.22	0.22	GGG	0.09	-0.54	TCA	-0.03	0.01	ATT	-0.31	0.14
GGC	0.21	0.38	AGA	0.08	0.07	GGT	-0.04	-0.30	TAT	-0.35	-0.33
GGA	0.19	0.38	CCT	0.06	0.13	TAC	-0.06	-0.14	ATA	-0.37	-0.05
CCC	0.19	-0.16	CAA	0.06	-0.11	ACT	-0.06	-0.13	TAG	-0.38	-0.56
AGC	0.18	0.04	TCC	0.06	0.01	CAT	-0.08	-0.03	TAA	-0.40	0.34
ACC	0.16	0.04	CAC	0.05	-0.18	AGT	-0.10	-0.10	TTA	-0.41	-0.39
CAG	0.15	0.15	AGG	0.05	-0.01	TTC	-0.11	0.38	TTT	-0.47	-0.60

The triplets are sorted based on their CP value starting from the maximal one.

## Testing of SP values of splicing enhancers and silencers

Tables 2, 3, 4, 5 present cumulative SP values for a number of known ESE and ESS sequences, calculated by summing the SP values of all triplets composing them. Since the ESE and ESS could have different lengths, we also calculated the average SP value per triplet. For example, the first motif in Table 2, *aggacagagc*, is composed of eight triplets (*agg, gga, gac, aca, cag, aga, gag, agc*). The sum of the SP values of these triplets is 0.89 and the value per triplet is 0.89/8 = 0.11.

**Table 2:** SP values of experimentally-determined exonic splicing enhancers.

No	ESE	Cum SP value	SP per triplet	SR protein
1	aggacagagc	0.89	0.11	ASF/SF2
2	aggacgaagc	1.71	0.21	ASF/SF2
3	rgaagaac	0.97	0.16	ASF/SF2
4	acgcgca	1.04	0.21	ASF/SF2
5	cctcgtcc	1.13	0.19	SRp20
6	acgaggay	1.39	0.23	9G8
7	aggagat	0.85	0.17	SC35

**Table 3:** SP values of RESCUE-predicted exonic splicing enhancers.

No	RESCUE-ESE	Cum SP	SP per triplet
1	atcttc	0.27	0.07
2	actaca	-0.43	-0.11
3	ttggat	0.60	0.15
4	gaatca	0.30	0.08
5	gaagaa	0.69	0.17
6	ttcaga	0.62	0.15
7	gacaaa	-0.07	-0.02
8	ctgaag	0.38	0.10
9	aatcca	0.09	0.02
10	aacttc	0.20	0.05

**Table 4:** SP values of experimentally-determined exonic splicing silencers.

No	Name	ESS	Cum SP value	SP per triplet	Core Cum SP value
1	R0624	agatcc**tagactaga**gcct	-0.12	-0.01	-1.31
2	R0628	ccaagt**caaaatttac**	-1.09	-0.08	-1.12
3	R0629	**tgtggg**	-0.86	-0.22	-0.86
4	R0632	agatccattcga**ttagtga**a	1.18	0.06	-1.23
5	R0634	ta**agtgtt**ctgagct	0.34	0.03	-0.82

**Table 5:** SP values of in vitro selected exonic splicing silencers.

Name	ESS	Cum SP value	SP per triplet
ESS1	TTTGTTCCGT	-0.77	-0.10
ESS2	GGGTGGTTTA	-2.28	-0.28
ESS3	GTAGGTAGGT	-2.16	-.270
ESS4	TTCGTTCTGC	1.32	0.16
ESS5	GGTAAGTAGG	-0.79	-0.10
ESS6	GGTTAGTTTA	-2.80	-0.35
ESS7	TTCGTAGGTA	-0.07	-0.01
ESS8	GGTCCACTAG	-1.22	-0.15
ESS9	TTCTGTTCCT	0.28	0.03
ESS10	TCGTTCCTTA	0.63	0.08
ESS11	GGGGTTGGGA	-1.80	-0.22
ESS12	GTTTGGGGGT	-2.77	-0.35
ESS13	TATAGGGGGG	-3.10	-0.39
ESS14	GGGGTTGGGA	-1.80	-0.22
ESS15	TTTCCTGATG	-0.01	0.00
ESS16	TGTTTAGTTA	-2.91	-0.36
ESS17	TTCTTAGTTA	-1.43	-0.18
ESS18	GTAGGTTTG	-2.10	-0.30
ESS19	GTTAGGTATA	-2.08	-0.26
ESS20	TAATAGTTTA	-1.51	-0.19
ESS21	TTCGTTTGGG	-0.17	-0.02

Table 2 presents a list of experimentally-determined non-redundant ESE sequences that have also been evaluated by another computational approach in Down et al. [9]. We do not consider ESEs with ambiguous bases in their internal regions (for instance, *tgcngyy *sequence) because even a single nucleotide substitution in the analyzed motif could dramatically change its cumulative SP value. All ESEs in Table 2 have high, positive, cumulative SP values. Their average cumulative SP value per triplet is 0.17.

Table 3 presents the consensus sequences of computer-predicted and verified ESE motifs obtained with the RESCUE-ESE method [11]. Eight out of ten of these RESCUE-ESE sequences also have positive cumulative SP values. Yet, the average SP per triplet (0.07) of these ten RESCUE-ESEs is much less than that of the experimentally-determined ESEs (0.17). Two out of ten RESCUE-ESE sequences have negative SP values (motifs #2 and #7, Table 3). Through the use of a different computational approach utilizing a machine learning strategy, these two motifs have also been shown to insignificantly impact splicing, or have "negative status," according to Down, et al. [9]. We also processed the total list of 238 putative RESCUE-ESE human splicing enhancers from the Hollywood exon annotation database [19]. The average SP value per triplet for this list is 0.08. 200 ESEs from this list have positive cumulative SP values and 38 are negative.

Table 4 presents experimentally verified ESS sequences from the RegRNA database [20]. We do not include very long (>50 nt) and very short (<5 nt) ESSs. We also excluded a controversial *GAAGAAGA *silencer motif because it overlaps with the well-known ESE motif *AAGAA*, as well as ESE #5 from Table 3. Table 4 demonstrates that three out of five of these ESSs have negative cumulative SP values. However, all ESSs from this list have core sequences (shown in bold) with highly negative cumulative SP values (shown in the last column in Table 4).

Finally, Table 5 presents a list of 21 *in vitro *selected putative ESSs published by Wang *et al*. [10]. Eighteen ESSs from this table have negative SP values, while two sequences have slightly positive SP values and only one sequence (ESS4) stands apart with a highly positive SP value (0.16 per triplet). The average cumulative SP for this group of 21 putative ESS is -0.17.

All in all we see a strong tendency for ESE to have positive cumulative SP-values and for core sequences of ESS to have negative cumulative SP-values. Therefore, this approach could be used for evaluation of a broad range of sequences for their contribution to the pre-mRNA splicing process.

## Testing the ability of SP to distinguish exons and introns

**Figure 1:**
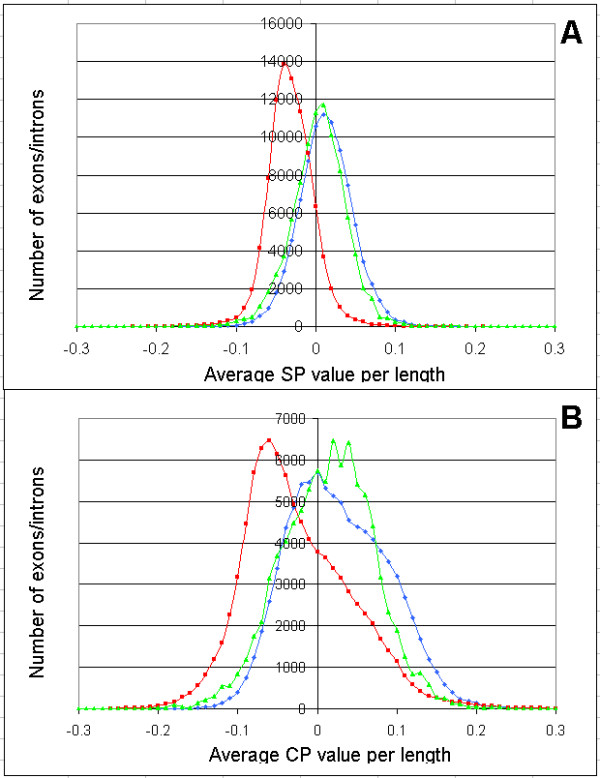
**Distribution of exons and introns by average SP values (A) and average CP values (B)**. Values were calculated for 90,178 constitutive human exons (blue curve) and the same number of introns form non-redundant human gene sample (red). Values for 9,768 skipped exons (green curves) were normalized to the number of constitutive exons exons (multiplied by 90178/9768 ratio). Vertical axis – number of exons or introns with particular average SP or CP values.

The capability of the SP and CP to distinguish between exons and introns has been examined. The complete sets of triplets composing each single exon and intron have been obtained (a sequence of *L *nucleotides is represented by (*L*-2) triplets). The average SP and CP values of exons and introns were calculated by summing the all triplet values and dividing by the number of triplets. The distributions of average SP and CP values per length for exons (red curve) and introns (blue curve) are shown in Fig. 1.

The overlapping area of the peaks represented by exon (blue) and intron (red) curves from Fig. 1 is 1.5 times smaller for the average SP values (46% overlap) than for the average CP values (68% overlap). Moreover, the SP values are significantly less variable than the CP values, which enhances the discriminating ability of SP in statistical tests (such as the t-test).

We also examined the distribution of average SP and CP values in the alternatively spliced exons of humans. Fig. 1 shows the distribution of average SP and CP values in a special case of alternative splicing – skipped exons with high skipping/retaining ratio (shown as green curves). Fig. 1A demonstrates that average SP values of skipped exons is very similar to constitutive exons (95% of curves overlapping), yet the curve for skipped exons has a slight, consistent shift toward the intron curve for every data point. The corresponding data for average CP-value curves (Fig. 1B) are not as smooth. There are several intersections between the average CP curves for skipped and constitutive exons. Thus the CP data is less amenable to interpretation.

## Discussion

Splicing Potential is a statistical approach for evaluating the involvement of oligonucleotides in splicing that is based solely on the ASMD dataset. HIt For each mutation we study the entire group of triplets overlapping this mutation because we do not know their individual contributions to splicing. Plausibly, a number of the triplets in these groups have no significant effect on splicing. These sequences produce statistical "noise," appearing in our processing algorithm in one set of instances as splicing enhancers (having positive SP_i _values) and in other cases as splicing silencers (with negative SP_i _values). Collecting more data on splicing mutations should statistically resolve such irrelevant oligonucleotides, bringing their SP values closer to zero.

Enlarging the ASMD dataset will present the opportunity to compute the SP values for larger oligonucleotides. To generate a reliable SP table for 4-mer nucleotides we need to know at least 250 mutations that affect splicing; for 5-mers, 800 mutations; and for 6-mers, at least 3000 mutations. It is well known that the predictive power of the coding potential (CP) increases dramatically with longer oligonucleotides: (*n*+1)-mers are always much better than *n*-mers, and 6-mers are the most commonly used oligonucleotides in real-world computations [21]. By analogy, we expect that the predictive power of SP will dramatically increase when SP values for longer oligonucleotides (up to 6-mers) have been computed.

We currently operate with the small set of 115 mutations in the ASMD. Even this limited dataset demonstrated an impressive trend in distinguishing between exonic and intronic sequences and also a very small, yet consistent, difference between constitutive and skipping exons. The SP values of triplets obtained on only 115 mutations is 1.5 times better at the separation of exons and introns compared to the analysis of triplet frequencies using Coding Potential. Further expansion of the ASMD dataset should dramatically increase the accuracy of the SP values and add power to this new tool for the prediction of exon/intron gene structures and, hopefully, alternative splicing.

Supplementary Methods can be found in Additional file1.

## Availability and requirements

**Project name: **Splicing Potential

**Project home page: **

**Operating system(s): **Platform-independent

**Programming Language: **Perl

**Other requirements: **a Perl 5 interpreter

**License: **GNU GPL v3

**Restrictions to use by non-academics: **None (not applicable under GPL)

## Competing interests

The authors declare that they have no competing interests.

## Authors' contributions

The Splicing Potential algorithm was conceptualized and developed by JMB, PR, II, YD, PKM, QW, XW, KAA, WEG, YW, and AF. SK was responsible for all statistical analyses. AF supervised the project, provided guidance, and wrote the draft. All authors have read and approved the final manuscript.

## Supplementary Material

Additional file 1 Supplementary Methods. Click here for file
