# Supplementary material for: Calculation of Splicing Potential from the Alternative Splicing Mutation Database
Source: BMC Res Notes. 2008 Feb 26;1:4. doi: 10.1186/1756-0500-1-4 (PMC2518266; doi:10.1186/1756-0500-1-4)
Supplement: Additional file 1 — Supplementary Methods. [file 1756-0500-1-4-S1.doc]

# MATERIALS AND METHODS (Supplementary file)

## Samples of exons and introns

Samples of 90,178 constitutively expressed human exons, as well as 9,567 and 31,414 skipped human exons with high and low frequency of appearance, respectively, were kindly provided by Sandro deSouza and Noboru Sakabe of the Ludwig Institute for Cancer Research, Sao Paulo, Brazil [22]. These samples are available upon personal request. The sample of 90,178 human introns is composed of the introns from our purged sample of 11,316 non-redundant human genes “HS35.1.purge3.dEID”. This latter sample is available from our web page (http://hsc.utoledo.edu/depts/bioinfo/asmd/). It was prepared based on 20,433 sequences of human intron-containing protein coding genes from the Exon-Intron Database [23]. This initial sample was purged by removing all homologs (≥50% protein identity) and genes with multiple repetitive domains (more than 4 repeats of the same 5-aa fragment) to obtain the reduced set of 11,316 human genes.

CP was calculated according to the formula (2) for the sample of 11,316 non-redundant human genes. In these computations F_c_(*x*) is the frequency of the triplet *x* in the coding sequences of the sample, and F_i_(*x*) is the frequency of the triplet *x* inside all introns.

## Programs

The “SplicingPotential.pl” program, its supporting documentation, and the associated files containing the results are available from http://hsc.utoledo.edu/depts/bioinfo/asmd/. The “SplicingPotential.pl” program takes as input the FASTA-formatted ASMD sequence data, and produces SP table and SP control files. The latter show all processing steps for each oligonucleotide (SP_i_ values). The computation of average SP and CP values was performed by the Perl program “AveragePotential.pl”, which is available from the same web location.
